# Whole‐brain deuterium metabolic imaging via concentric ring trajectory readout enables assessment of regional variations in neuronal glucose metabolism

**DOI:** 10.1002/hbm.26686

**Published:** 2024-04-22

**Authors:** Fabian Niess, Bernhard Strasser, Lukas Hingerl, Viola Bader, Sabina Frese, William T. Clarke, Anna Duguid, Eva Niess, Stanislav Motyka, Martin Krššák, Siegfried Trattnig, Thomas Scherer, Rupert Lanzenberger, Wolfgang Bogner

**Affiliations:** ^1^ High Field MR Center, Department of Biomedical Imaging and Image‐Guided Therapy Medical University of Vienna Vienna Austria; ^2^ Wellcome Centre for Integrative Neuroimaging, FMRIB, Nuffield Department of Clinical Neurosciences University of Oxford Oxford UK; ^3^ Christian Doppler Laboratory for MR Imaging Biomarkers (BIOMAK), Department of Biomedical Imaging and Image‐guided Therapy Medical University of Vienna Vienna Austria; ^4^ Department of Medicine III, Division of Endocrinology and Metabolism Medical University of Vienna Vienna Austria; ^5^ Institute for Clinical Molecular MRI Karl Landsteiner Society St. Pölten Austria; ^6^ Department of Psychiatry and Psychotherapy, Comprehensive Center for Clinical Neurosciences and Mental Health (C3NMH) Medical University of Vienna Vienna Austria

**Keywords:** 3D magnetic resonance spectroscopic imaging, deuterium metabolic imaging, deuterium‐labeled glucose, downstream neurotransmitter synthesis, whole‐brain metabolic mapping

## Abstract

Deuterium metabolic imaging (DMI) is an emerging magnetic resonance technique, for non‐invasive mapping of human brain glucose metabolism following oral or intravenous administration of deuterium‐labeled glucose. Regional differences in glucose metabolism can be observed in various brain pathologies, such as Alzheimer's disease, cancer, epilepsy or schizophrenia, but the achievable spatial resolution of conventional phase‐encoded DMI methods is limited due to prolonged acquisition times rendering submilliliter isotropic spatial resolution for dynamic whole brain DMI not feasible. The purpose of this study was to implement non‐Cartesian spatial‐spectral sampling schemes for whole‐brain ^2^H FID‐MR Spectroscopic Imaging to assess time‐resolved metabolic maps with sufficient spatial resolution to reliably detect metabolic differences between healthy gray and white matter regions. Results were compared with lower‐resolution DMI maps, conventionally acquired within the same session. Six healthy volunteers (4 m/2 f) were scanned for ~90 min after administration of 0.8 g/kg oral [6,6′]‐^2^H glucose. Time‐resolved whole brain ^2^H FID‐DMI maps of glucose (Glc) and glutamate + glutamine (Glx) were acquired with 0.75 and 2 mL isotropic spatial resolution using density‐weighted concentric ring trajectory (CRT) and conventional phase encoding (PE) readout, respectively, at 7 T. To minimize the effect of decreased signal‐to‐noise ratios associated with smaller voxels, low‐rank denoising of the spatiotemporal data was performed during reconstruction. Sixty‐three minutes after oral tracer uptake three‐dimensional (3D) CRT‐DMI maps featured 19% higher (*p* = .006) deuterium‐labeled Glc concentrations in GM (1.98 ± 0.43 mM) compared with WM (1.66 ± 0.36 mM) dominated regions, across all volunteers. Similarly, 48% higher (*p* = .01) ^2^H‐Glx concentrations were observed in GM (2.21 ± 0.44 mM) compared with WM (1.49 ± 0.20 mM). Low‐resolution PE‐DMI maps acquired 70 min after tracer uptake featured smaller regional differences between GM‐ and WM‐dominated areas for ^2^H‐Glc concentrations with 2.00 ± 0.35 mM and 1.71 ± 0.31 mM, respectively (+16%; *p* = .045), while no regional differences were observed for ^2^H‐Glx concentrations. In this study, we successfully implemented 3D FID‐MRSI with fast CRT encoding for dynamic whole‐brain DMI at 7 T with 2.5‐fold increased spatial resolution compared with conventional whole‐brain phase encoded (PE) DMI to visualize regional metabolic differences. The faster metabolic activity represented by 48% higher Glx concentrations was observed in GM‐ compared with WM‐dominated regions, which could not be reproduced using whole‐brain DMI with the low spatial resolution protocol. Improved assessment of regional pathologic alterations using a fully non‐invasive imaging method is of high clinical relevance and could push DMI one step toward clinical applications.

## Graphical abstract

After oral administration of deuterium‐labeled glucose, ten 3D metabolic datasets were acquired over ∼70 min using 2 mL (orange) and 0.75 mL (blue) isotropic spatial resolution. High‐resolution metabolic maps featured 48% higher (*p* < .01) glutamate + glutamine in GM compared with WM regions, while no contrast was observed in low‐resolution maps (*p* = .52).

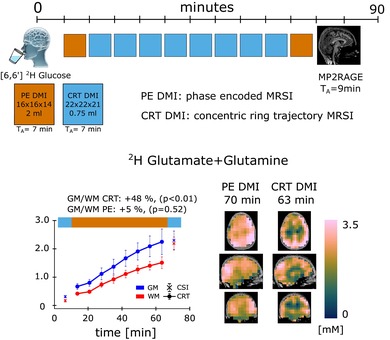

**Practitioner Points**

- 2.5‐fold increased spatial resolution was achieved compared with conventional whole‐brain DMI maps
- High‐resolution CRT‐DMI maps allowed to visualize increased contrast in metabolic activity (+48% glutamate + glutamine, +19% glucose) in GM compared with WM dominated regions
- Low‐resolution PE‐DMI maps revealed lower contrast in metabolic activity for Glc (+16%) and no differences for Glx between GM and WM regions.

## 1 INTRODUCTION

Glucose serves as a primary source to produce energy in the form of adenosine triphosphate (ATP) in the mammalian brain, which is essential for maintaining neuronal activity, neurotransmitter release and brain cell communication (Dienel, 2019; Magistretti & Allaman, 2015). An improved understanding of brain glucose metabolism could contribute to advancements in neuroscience and aid the development of targeted interventions for various pathologic conditions, such as neurodegenerative disorders, epilepsy, psychiatric disorders and brain tumors (Koppenol et al., 2011; Manji et al., 2012; Norat et al., 2020).

The current clinical gold standard to image tissue‐specific glucose uptake is positron emission tomography (PET), using Fluorodeoxyglucose ([^18^F]‐FDG) as intravenous, and radioactive tracer. However, due to glucose trapping of the tracer, PET does not provide information about downstream metabolites, for example, oxidatively synthesized glutamate or glycolytically produced lactate (Almuhaideb et al., 2011; Hahn et al., 2016).

Deuterium metabolic imaging (DMI) (De Feyter et al., 2018; Lu et al., 2017; Ruhm et al., 2021; Seres Roig et al., 2023; Veltien et al., 2021) and quantitative exchange label turnover (QELT) (Bednarik et al., 2023; Niess, Hingerl, et al., 2023; Rich et al., 2020; Ruhm et al., 2022) are novel magnetic resonance techniques to non‐invasively image glucose metabolism using direct (^2^H MRSI) and indirect (^1^H MRSI) detection of administered deuterium‐enriched substrates, respectively. QELT does not require additional non‐proton RF hardware and features higher sensitivity, which allows for higher spatial resolution compared with DMI. However, lipid contaminations from subcutaneous fat outside of the brain and motion sensitivity impose challenges during signal acquisition and image reconstruction.

Due to a 6.5‐fold lower gyromagnetic ratio compared with hydrogen DMI applications feature a more homogeneous distribution of the static magnetic field (B_0_) and the transmit field (B_1_) even at ultra‐high magnetic field strengths >7 T. Additionally, due to low natural abundance of deuterium (0.015%), DMI features spectral sparsity and does not require dedicated water or lipid suppression methods, while the water resonance can be used as internal reference for concentration estimation.

The achievable spatial resolution of conventional DMI methods is limited due to prolonged acquisition times of Cartesian phase‐encoding readout, rendering submilliliter isotropic spatial resolution for dynamic whole brain DMI not feasible. Significant acceleration factors for magnetic resonance spectroscopic imaging (MRSI) have been achieved using spatial‐spectral sampling schemes, which allow for substantially increased spatial resolution (Furuyama et al., 2012; Hingerl et al., 2020; Jiang et al., 2016; Maudsley et al., 2009). Sufficiently high spatial resolution is crucial not only for anatomical imaging modalities to characterize abnormalities in biological structures on a fine scale, but also for metabolic imaging to reliably detect local pathologic alterations of the metabolism in several brain diseases, for example, multiple sclerosis (Heckova et al., 2019, 2022; Lipka et al., 2023) or brain tumors (Hangel et al., 2020).

Faster metabolic activity has been reported for gray matter compared with white matter brain tissue, represented by higher glucose uptake detected via PET (Yu et al., 2018), higher TCA cycle rates estimated using ^13^C‐MRS (Pan et al., 2000) and faster dynamics of glutamate + glutamine synthesis detected via indirect deuterium detection using ^1^H MRSI (Bednarik et al., 2023; Niess, Strasser, et al., 2023).

The purpose of this study was to implement spatial‐spectral sampling for whole‐brain ^2^H FID‐MRSI to obtain time‐resolved metabolic maps with increased spatial resolution. To assess the expected improvements metabolic differences of glucose metabolism between gray‐ and white matter‐dominated regions were quantified. The results were compared with conventional DMI maps, which were acquired within the same session, but with lower spatial resolution using phase encoding readout.

## 2 MATERIALS AND METHODS

### 2.1 Study participants

Six healthy volunteers (age: 26 ± 2 years; BMI: 23 ± 4 kg/m^2^, 4 male/2 female) participated in the study after written informed consent was obtained. The study was approved by the local ethics committee of the Medical University of Vienna according to the guidelines of the Declaration of Helsinki. Participants were scanned after overnight fasting in the morning and immediately after oral administration of deuterium‐labeled glucose (0.8 g/kg body weight, [6,6′]‐^2^H‐Glc ≥99% purity, Cambridge Isotopes) dissolved in ∼200 mL water. The tracer was consumed in ∼1 min, directly on the patient table of the MR scanner, immediately before volunteers were moved inside the magnet bore.

### 2.2 Deuterium metabolic imaging protocol

**FIGURE 1:**
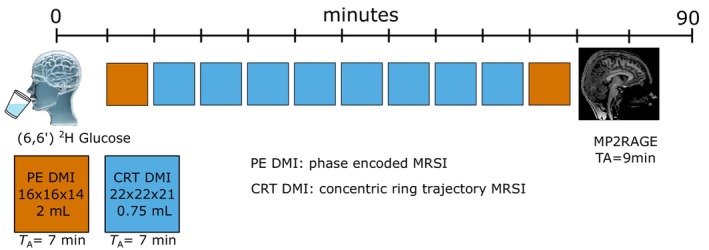
Schematic illustration of the experimental protocol. After oral administration of [6,6′]‐^2^H Glucose the subject is placed into the magnet bore. Following initial preparation, ten 3D deuterium metabolic imaging (DMI) datasets were acquired dynamically over the course of ∼70 min using two sampling schemes with matched acquisition duration, that is, elliptical phase encoding (PE) with 2 mL isotropic resolution (orange) and concentric ring trajectories (CRT) with 0.75 mL (blue). High resolution anatomical images were acquired using MP2RAGE scans.

All measurements were performed on a human whole‐body 7 T (Magnetom dot Plus) Siemens MR system using a ^2^H/^1^H dual‐tuned quadrature birdcage head coil (Stark Contrasts MRI Coils Research, Germany). No modification of the scanner hardware or software was required as the acquisition of ^2^H signals are supported by the vendor. Following initial preparations scans, that is, auto‐align localizer images and unlocalized pulse‐acquire B_1_ estimation (*T*_R_ = 1.5 s, *T*_E_ = 0.35 ms, 20 steps, *U*_Ref_ = 20–440 V), and ∼7 min after oral glucose uptake, ten whole brain three‐dimensional (3D) DMI datasets of deuterium labeled (^2^H) water, glucose (Glc) and glutamate + glutamine (Glx) were consecutively measured over the course of ∼70 min (∼7 min per 3D dataset) see Figure 1.

Two 3D DMI datasets were acquired with lower spatial resolution (first and tenth time point, 7 and 70 min after glucose uptake, respectively) similar to (Niess, Strasser, et al., 2023) using elliptical phase encoding (PE‐DMI) with ∼2 mL isotropic nominal voxel size with the following parameters: *T*_R_ = 290 ms, acquisition delay: 1.56 ms, non‐localized rectangular excitation pulse (flip angle: 86°, pulse duration: 500 μs), matrix: 16 × 16 × 14, FOV: 200 × 200 × 175 mm^3^, bandwidth: 500 Hz, number of samples: 128.

Eight datasets (second to ninth time point, 14–63 min after tracer uptake) were acquired using a previously developed ^2^H FID MRSI (Hingerl et al., 2020) sequence using 3D density‐weighted concentric ring trajectory readout (CRT‐DMI) (Chiew et al., 2018; Clarke et al., 2023; Hingerl et al., 2018; Steel et al., 2018) with ∼0.75 mL isotropic spatial resolution (identical excitation pulse as for PE‐DMI, *T*_R_ = 290 ms, acquisition delay: 2.0 ms, matrix: 22 × 22 × 21, FOV: 200 × 200 × 192 mm^3^, *N*_circles_ = 43, bandwidth 380 Hz, number of samples 96). No zero‐filling was applied. After the DMI acquisitions a high‐resolution T_1_ weighted anatomical image was acquired (MP2RAGE) using the following parameters: FOV: 165 × 220 × 220 mm^3^, matrix: 144 × 192 × 192, *T*_R_ = 3930 ms, *T*_I1_ = 850 ms, *T*_I2_ = 3400 ms, *T*_E_ = 3.28 ms, *T*_A_ = 9.29 min. The total duration of the DMI protocol was 86 min. For detailed information about sequence parameters see Table S1 for minimum reporting standards (Lin et al., 2021).

### 2.3 Data processing and metabolite quantification

Image reconstruction and spectral fitting of deuterium‐labeled metabolite resonances was performed offline using an in‐house developed post‐processing pipelines (MATLAB R2021, LCModel v6.3, Python3.10). Cartesian phase‐encoded (PE) DMI data were Hamming filtered in all three spatial dimensions followed by a 3D Fourier transform.

DMI data acquired using CRT‐DMI were reconstructed using non‐Cartesian 3D discrete Fourier transformation without density compensation (Clarke et al., 2023; Hingerl et al., 2018). For spectral fitting of DMI data a basis set was simulated (Naressi et al., 2001; Starcuk et al., 2009) including ^2^H resonances of “natural abundance” deuterium water (4.8 ppm), Glc (3.9 ppm), Glx (2.4 ppm) and lactate/fat (1.3 ppm). This work focuses only on water, Glc and Glx maps. Quantification results with Cramer–Rao Lower Bounds (CRLB) >50% were excluded from further analysis.

### 2.4 Segmentation

Tissue segmentation of gray (GM) and white matter (WM) regions was performed on high‐resolution T_1_‐weighted anatomical 3D images (MP2RAGE) by calculating voxel‐wise fraction maps of GM, WM and CSF using the FAST algorithm (Jenkinson et al., 2012), followed by down‐sampling to MRSI grid‐size in the k‐space considering partial volume effects of the 3D Hamming filter. For regional averaging over GM‐ and WM‐dominated regions a 55% and 60% threshold were used for low‐resolution PE‐DMI and higher‐resolution CRT‐DMI, respectively.

### 2.5 Concentration estimation

The 3D maps of ^2^H Glc and Glx concentration estimates (in mM) were created for all time points. Voxel‐wise concentration estimation was performed using natural abundance water maps from the beginning of the DMI protocol (first and second time point for PE‐DMI and CRT‐DMI, respectively) as internal reference according to (Gasparovic et al., 2006) assuming relaxation times and in vivo concentrations from literature (Cocking et al., 2023; Seres Roig et al., 2023) (^2^H:water: *T*_1GM_/*T*_1WM_/*T*_1CSF_ = 320/290/510 ms, *T*_2GM/WM_/*T*_2CSF_ = 30/90 ms, Glx: *T*_1_/*T*_2_ = 149/53 ms, Glc: *T*_1_/*T*_2_ = 66/44 ms, ^2^H water concentration: 17.2 mM). The number of deuterium atoms per molecule was accounted for, see Appendix 1. Voxel‐wise fractional water content of GM, WM and CSF was taken into account, from downsampled segmentation maps. DMI maps of metabolized Glx were corrected for an assumed 40% label loss in the Tricarboxylic acid cycle (TCA), which is in agreement with recent literature (de Graaf et al., 2021).

### 2.6 Comparison between PE‐DMI and CRT‐DMI

Voxel‐wise signal‐to‐noise ratios (SNR) were calculated for both PE‐DMI and CRT‐DMI acquisition methods using the amplitude of natural abundance water and the standard deviation of the noise in a region ∼100 Hz off‐resonant from water. Metabolite concentrations of Glc and Glx were compared between PE‐DMI and CRT‐DMI maps, measured at 63 and 70 min after oral tracer uptake, respectively. Additionally, Glc and Glx concentrations were averaged for each time point over GM and WM dominated regions, across all volunteers to investigate the dynamics of glucose metabolism.

### 2.7 Denoising

CRT‐DMI features higher spatial resolution, i.e., the nominal voxel volume is ∼2.5‐fold smaller compared with PE‐DMI, consequently decreasing overall SNR. To mitigate the effective loss of SNR, all data were reshaped into a modified 2D structure (see Appendix 2) and global low‐rank spatiotemporal denoising (rank = 8) was applied to all reconstructed time domain CRT‐DMI data using singular value decomposition (MATLAB R2021).

The performance and reliability of the denoising algorithm to correctly estimate signal concentrations in denoised spectra was verified on synthetic data mimicking the data shape, quality and dynamics of the CRT‐DMI protocol acquired in vivo. The accuracy and precision of the resulting de‐noised metabolic maps were compared with the gold standard (based on the known ground truth of noiseless spectra).

### 2.8 Synthetic data

High‐resolution synthetic brain MRSI datasets (220 × 220 × 210 voxels, 96 spectral points and 8 time points) were created, consisting of simulated deuterium spectra (^2^H resonances: water, Glc and Glx) with and without random Gaussian noise added. While constant water concentrations were generated over all time points, Glc and Glx concentrations were introduced to increase over time, monoexponentially (time constant = 15 min) and linearly, respectively. Additionally, a regional contrast was introduced with higher concentrations in synthetic gray matter (sGM) compared with synthetic white matter (sWM), that is, 20%, 33% and 66% for water, Glc and Glx, respectively (Gasparovic et al., 2006). Datasets were then down‐sampled to match CRT‐DMI protocol parameters, (i.e., matrix size: 22 × 22 × 21) considering effects of the point spread function and partial volume contamination due to Hamming weighting. Masks for segmentation of sGM and sWM voxels were created in similar fashion. Low‐rank denoising (rank = 8) was performed on the synthetic dataset with added noise. Overall, three synthetic datasets were used as input for spectral fitting using LCModel: no noise (GS, defined as gold standard), added noise (NO) and low‐rank denoised (LR). Spectral fitting of the synthetic data was performed using the same post processing pipeline as for in vivo data. Following spectral fitting, metabolic maps of GS, NO and LR were regionally averaged over sGM and sWM using down‐sampled masks (threshold >70%) for each time point. Time courses of increasing Glc and Glx concentrations were fitted mono‐exponentially and linearly, respectively. Dynamic fitting results of Glc and Glx concentration were compared between GS, NO and LR datasets and regional differences were analyzed for the last time point.

### 2.9 Statistical analysis

Pearson correlation analysis was performed between regionally averaged PE‐DMI and CRT‐DMI metabolite concentrations (Glc and Glx) and time. Due to the relatively small sample size a Student *t*‐tests with a statistical significance threshold of *p* < .05 was used to estimate differences between groups (de Winter, 2013). The Benjamini and Hochberg method was used to adjust *p*‐values for correction of multiple testing (Jafari & Ansari‐Pour, 2019). Statistical tests were performed using Python 3.10 (www.python.org, packages: scipy. stats). For metabolic maps the “batlow” color map was used as recommended in Crameri et al. (2020).

## 3 RESULTS

### 3.1 Deuterium metabolic imaging protocol

Following oral administration of deuterium labeled glucose initial preparation scans including auto‐align localizer, B_1_ adjustment and manual shimming were performed in 8 ± 1 min. The DMI protocol consisting of 10 consecutive 3D metabolic data acquisitions using two different spatial resolutions (2 vs. 0.75 mL) was successfully performed in all six volunteers, without complications or volunteer dropouts. The standardized quality criteria used for data exclusion were fulfilled by more than 90% of voxels for both acquisition schemes. The SNR was 27 ± 2 in PE‐DMI maps and 12 ± 1 in CRT‐DMI maps, across all volunteers. The average linewidth of the natural abundance water resonance (real part) from the first time point was 12.1 ± 0.9 Hz and 12.2 ± 0.6 Hz for PE‐DMI and CRT‐DMI, respectively. Following low‐rank denoising of CRT‐DMI data, the SNR increased (*p* < .001) to 34 ± 4, across all volunteers.

**FIGURE 2:**
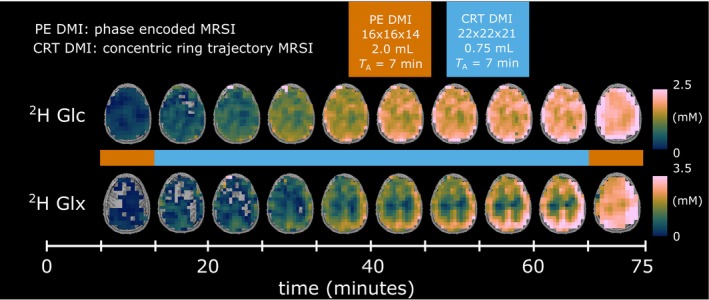
Time courses of axial ^2^H glucose (Glc) and ^2^H glutamate + glutamine (Glx) maps given in mM from one representative volunteer, detected using deuterium metabolic imaging (DMI) with phase encoded readout (orange) and concentric ring trajectory readout (blue) at 7 T. Missing voxels in the metabolic maps do not contain a value. NaN, not a number.

Time courses of axial metabolic maps of ^2^H‐glucose (Glc) and ^2^H‐glutamate + glutamine (Glx) from one representative volunteer are shown in Figure 2, while individual maps from all participants are shown in Figures S1 and S2.

**FIGURE 3:**
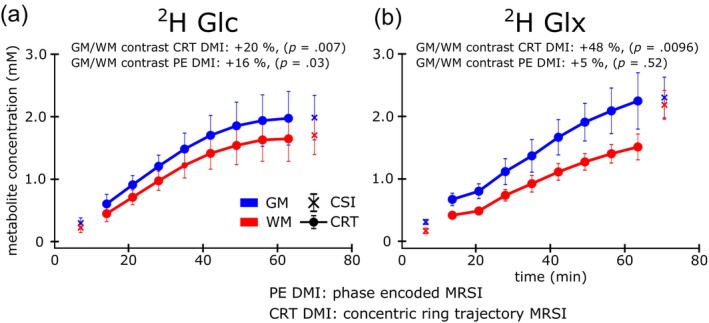
Time courses of ^2^H labeled glucose (Glc, a) and glutamate + glutamine (Glx, b) and regionally averaged over gray (GM) and white matter (WM) and over all subjects. Significant differences between GM and WM were observed for higher spatial resolution acquisitions for deuterium metabolic imaging using concentric ring trajectory readout (CRT DMI, 0.75 mL voxel volume) with 20% higher (*p* = .007) Glc and 48% higher (*p* = .0096) Glx observed in GM compared with WM. Lower spatial resolution scans using phase encoded DMI (PE DMI, 2 mL voxel volume) showed smaller differences between GM and WM for Glc (+16%, *p* = .03), while no differences were found for Glx (*p* = .52).

Time courses of ^2^H‐Glc and ^2^H‐Glx regionally averaged over gray (GM) and white matter (WM) dominated regions and over all subjects are illustrated for both PE‐DMI and CRT‐DMI scans in Figure 3.

### 3.2 Concentric ring trajectory deuterium metabolic imaging

Sixty‐three minutes after oral administration of deuterium labeled [6,6′]‐Glucose ^2^H‐Glc concentrations in concentric ring trajectory deuterium metabolic imaging (CRT‐DMI) maps increased to 1.99 ± 0.43 mM (*p* = .0063) and 1.66 ± 0.36 mM (*p* = .0056) in GM and WM dominated regions, respectively, over all volunteers, see Figure 3a. Similarly, ^2^H Glx concentrations increased to 2.21 ± 0.44 mM (*p* = .003) and 1.49 ± 0.20 mM (*p* < .001) in GM and WM, respectively, see Figure 3b. Significantly higher ^2^H‐Glx concentrations were observed in GM compared with WM for ^2^H‐Glx (+48%, *p* = .0096), on average over all volunteers, representing faster metabolic activity. Smaller regional differences between GM and WM were observed for ^2^H‐Glc concentrations (+20%, *p* = .007).

Individual results of regionally averaged ^2^H‐Glc and ^2^H‐Glx concentrations acquired using CRT‐DMI (63 min) and PE‐DMI (70 min after tracer uptake) are shown in Table 1 for all volunteers.

**TABLE 1:** Individual concentrations for deuterium labeled glucose (Glc) and glutamate + glutamine (Glx) from gray (GM) and white matter (WM) dominated regions in the human brain, acquired 63 and 70 min after oral tracer uptake using phase encoded (PE) and concentric ring trajectory (CRT) deuterium metabolic imaging (DMI).

	Gray matter				White matter			
	Glx [mM]		Glc [mM]		Glx [mM]		Glc [mM]	
Volunteer	PE DMI	CRT DMI	PE DMI	CRT DMI	PE DMI	CRT DMI	PE DMI	CRT DMI
	(2 mL)	(0.75 mL)	(2 mL)	(0.75 mL)	(2 mL)	(0.75 mL)	(2 mL)	(0.75 mL)
No. 1	2.5 ± 1.4	2.4 ± 0.9	1.4 ± 0.7	1.3 ± 0.5	2.3 ± 0.6	1.7 ± 0.6	1.4 ± 0.4	1.2 ± 0.2
No. 2	2.3 ± 1.2	2.9 ± 0.7	2.2 ± 0.3	2.1 ± 0.3	2.4 ± 0.6	1.8 ± 0.6	1.7 ± 0.2	1.7 ± 0.3
No. 3	2.1 ± 1.3	1.9 ± 0.5	2.0 ± 0.4	2.0 ± 0.3	2.0 ± 0.4	1.3 ± 0.5	1.6 ± 0.1	1.5 ± 0.2
No. 4	1.7 ± 1.1	1.7 ± 0.5	2.4 ± 0.7	2.7 ± 0.5	1.8 ± 0.4	1.4 ± 0.5	2.2 ± 0.2	2.3 ± 0.3
No. 5	2.2 ± 1.0	1.8 ± 0.6	1,6 ± 0.4	1.6 ± 0.4	2.0 ± 0.5	1.2 ± 0.5	1.4 ± 0.2	1.3 ± 0.2
No. 6	2.7 ± 0.9	2.6 ± 0.7	2.4 ± 0.5	2.2 ± 0.4	2.4 ± 0.4	1.6 ± 0.5	2.0 ± 0.2	1.9 ± 0.3

*Note*: Spatial resolution in mL (2 mL for PE, 0.75 mL for CRT).

Abbreviations: CRT, concentric ring trajectory readout; DMI, deuterium metabolic imaging; Glc, Glucose; Glx, Glutamate + Glutamine; PE, phase encoding readout.

### 3.3 Phase encoded deuterium metabolic imaging (PE‐DMI)

Seventy minutes after oral [6,6′]‐^2^H‐Glc administration the concentrations of ^2^H‐Glc increased to 2.00 ± 0.36 mM (*p* < .001) and 1.71 ± 0.31 mM (*p* < .001) in GM and WM, respectively, see Figure 3a. Similarly, ^2^H‐Glx concentrations increased to 2.26 ± 0.32 mM (*p* < .001) and 2.15 ± 0.22 mM (*p* < .001) in GM‐ and WM‐dominated regions, respectively, see Figure 3b. Higher ^2^H‐Glc concentrations were observed in GM compared with WM regions (+16% *p* = .03). No regional differences were observed for ^2^H‐Glx (*p* = .52) concentrations.

### 3.4 CRT‐DMI versus PE‐DMI

**FIGURE 4:**
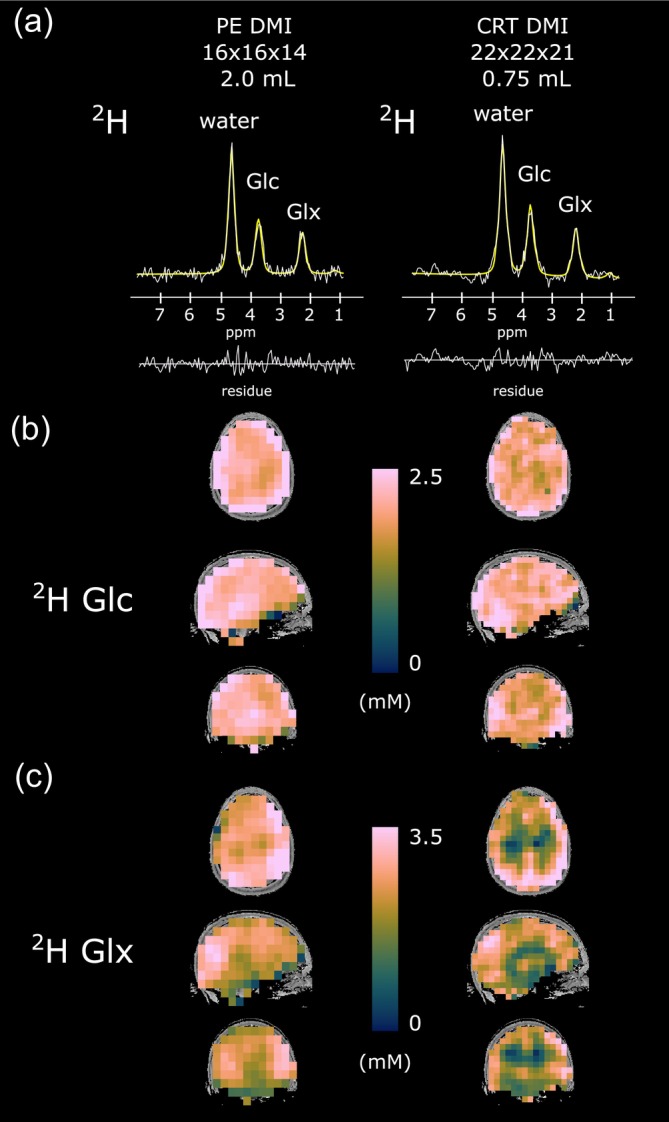
Comparison of spatial resolution between 2 mL and 0.75 mL for phase encoded deuterium metabolic imaging (PE DMI, left column) and concentric ring trajectory DMI (CRT DMI, right column), respectively. Representative sample spectra, spectral fits and residues (a) of the last DMI scan (63 and 70 min after oral administration of deuterium labeled glucose) featuring resonances of ^2^H water, ^2^H glucose (Glc) and ^2^H glutamate + glutamine (Glx). The 3D metabolic maps of Glc (b) and Glx (c) from one representative volunteer. Faster metabolism in GM compared with WM, that is, increased Glx concentrations are more pronounced for CRT maps.

A direct comparison between CRT‐DMI maps with 0.75 mL isotropic resolution and PE‐DMI maps with 2 mL isotropic voxel size to non‐invasively image glucose uptake and oxidative downstream neurotransmitter (Glx) synthesis in the human brain is illustrated in Figure 4 showing sample spectra, spectral fits and 3D metabolic maps (^2^H‐Glc and ^2^H‐Glx) from one representative volunteer, 63 min (CRT‐DMI) and 70 min (PE‐DMI) after oral glucose administration.

Averaged ^2^H‐Glc concentrations were not significantly different between CRT‐DMI (63 min) and PE‐DMI (70 min after tracer uptake) datasets, for both GM (*p* = .88) and WM (*p* = .54) dominated regions. While averaged ^2^H‐Glx concentrations were similar (*p* = .72) in GM dominated regions of CRT‐DMI and PE‐DMI maps, differences were observed in WM regions (*p* = .002).

**FIGURE 5:**
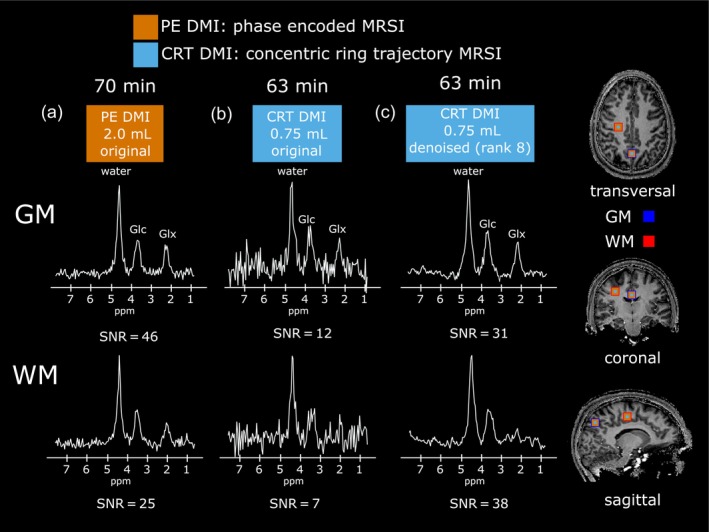
Representative sample spectra from a single predominantly gray matter (GM) and white matter (WM) voxel of one volunteer, using phase encoded deuterium metabolic imaging (orange, PE DMI, 2 mL isotropic voxel volume) (a) and concentric ring trajectory DMI (blue) before (b) and after denoising (c) using singular value decomposition with low rank estimation (rank = 8).

Sample spectra of representative GM and WM voxels for both PE‐DMI and CRT‐DMI (original and after low‐rank denoising) acquisition schemes are shown in Figure 5.

### 3.5 Low‐rank denoising of synthetic data

^2^H spectra and spectral fits of a single representative voxel from the last time point (63 min) for three matching synthetically created CRT‐DMI datasets: gold standard (GS), added noise (NO) and low‐rank de‐noised (LR) are shown in Figure S3a. Denoising increased the SNR (water amplitude/standard deviation of noise) ~3.5‐fold from 22 ± 7 (NO) to 78 ± 25 (LR) (*p* < .001) on average across all voxels and time points. Corresponding metabolic maps of synthetic ^2^H‐water, ^2^H‐Glc and ^2^H‐Glx, for GS, NO and LR datasets are illustrated in Figure S3b. Regionally higher ^2^H‐Glc concentrations in synthetic gray (sGM) compared with synthetic white matter (sWM) at the last time point were overall in good agreement between noise‐less GS (19%), NO (20%) and LR (23%) maps. Similarly, 43% higher ^2^H‐Glx concentrations in synthetic gray matter (sGM) compared with white matter (sWM) was overall in very good agreement, for NO maps (44%), and LR maps (42%). Static concentration differences of ^2^H‐water between sGM and sWM over all time points were closer approximated and not decreased in LR maps (9% ± 1%) compared with the gold standard (10%), while higher variation was observed for NO maps (9% ± 2%).

The voxel‐wise accuracy of NO maps compared with GS, showed differences of 11% ± 0.4%, 19% ± 6% and 42% ± 25% for ^2^H‐water, ^2^H‐Glc and ^2^H‐Glx concentrations, respectively, on average over all time points. After denoising the accuracy improved and differences compared with GS maps significantly decreased to 4% ± 0.5% (*p* < .001), 7% ± 2% (*p* = .003) and 14% ± 6% (*p* = .02) for ^2^H‐water, ^2^H‐Glc and ^2^H‐Glx, respectively.

Similarly, the precision of LR maps is higher compared with NO maps, reflected by significantly decreased coefficients of variation (COV) from 10% ± 1%, 17% ± 4%, and 40 ± 23% in NO maps to 7% ± 2% (*p* < .001), 9% ± 1% (*p* < .001) and 16% ± 5% (*p* = .004) in LR maps, for ^2^H‐water, ^2^H‐Glc and ^2^H‐Glx, respectively over all time points.

Time courses of, ^2^H metabolite concentrations, regionally averaged over sGM and sWM for GS, NO and LR are illustrated in Figure S4. Calculated exponential time constants of increasing ^2^H‐Glc concentrations and slopes of the linear fit of ^2^H‐Glx concentrations between NO, LR and GS were comparable, with higher variability in NO maps compared with LR maps, see Figure S4a,b.

## 4 DISCUSSION

This work presents time‐resolved non‐invasive 3D imaging of human brain glucose metabolism with ∼2.5‐fold increased spatial resolution compared with reported whole brain DMI maps acquired at comparable magnetic field strengths.

After oral administration of deuterium‐labeled glucose its uptake and downstream oxidative metabolism in the human brain could be assessed dynamically by acquiring whole‐brain metabolic maps of glucose (Glc) and combined glutamate + glutamine (Glx) with a submilliliter isotropic resolution (0.75 mL) consecutively every 7 min.

To fully understand the underlying metabolic model of brain glucose metabolism, real quantitative turnover rates and metabolic fluxes have to be derived, which require dynamic assessments of the glucose transport and utilization kinetics and additionally time resolved measurements of blood plasma glucose levels. While the dynamics of glucose metabolism in the brain were assessed via time‐resolved measurements of increasing ^2^H‐Glc and ^2^H‐Glx concentrations, metabolic modeling was not the aim of this study. Therefore, the study protocol of this work did not include invasive sampling of blood glucose levels. Simultaneous time resolved capillary puncture of the toe has been performed in previous studies (Bednarik et al., 2023; Niess, Hingerl, et al., 2023; Niess, Strasser, et al., 2023) to avoid interruption of the DMI protocol, but it was notoriously difficult, and required additional medical personnel. This study focused on analysis of the regional metabolic variation between gray and white matter tissue reflected by tissue specific Glc and Glx concentrations ~1 h after oral glucose uptake. Results were compared with metabolic maps acquired with lower spatial resolution (2 mL) using more commonly used 3D phase encoding readout (PE‐DMI) within the same study protocol.

Increasing concentrations of brain ^2^H‐Glc and ^2^H‐Glx were in good agreement with DMI studies using similar glucose administration (De Feyter et al., 2018; Heikkila et al., 2010; Ruhm et al., 2021) and inter‐subject variation was comparable with a previous study in a different cohort of subjects (Niess, Strasser, et al., 2023).

Sixty‐three minutes after tracer uptake regional differences were observed in ^2^H‐Glx maps acquired using CRT‐DMI, that is, +48% higher concentrations in gray matter (GM)‐ compared with white matter (WM)‐dominated regions representing faster metabolic activity.

Observed regional GM/WM variations of metabolic activity are in good agreement with values reported in [^18^F]FDG‐PET studies (Yu et al., 2018) (∼33% higher oxidative Glc consumption indirectly indicating faster metabolic activity) and recent work of our own group using indirect deuterium detection ((∼50% higher Glx concentrations in GM vs. WM) ^1^H MRSI) (Bednarik et al., 2023; Niess, Hingerl, et al., 2023; Niess, Strasser, et al., 2023), but lower than reported values of glutamate turnover rates detected with ^13^C MRS (difference of ∼68% between GM and WM) (de Graaf et al., 2001; Pan et al., 2000).

While steady‐state glucose concentrations during fasting glycemia and hyperglycemia detected via single voxel ^1^H‐MRS were reported to be similar between GM of the cerebral cortex and WM tissue (de Graaf et al., 2001; Hawkins et al., 1983; Pan et al., 2000), significant differences were reported for increasing hyperglycemic ^1^H‐Glc concentrations between cerebellum and WM regions (Heikkila et al., 2010).

This is in the range of our results, which show +20% higher ^2^H‐Glc concentrations observed in GM compared with WM regions, as GM segmentation was performed over the whole brain including the cerebellar cortex, consisting predominantly of GM tissue.

Regional differences for ^2^H‐Glx concentrations could not be reproduced using conventional 3D phase encoding readout (PE‐DMI) with lower resolution (2 mL), presumably caused by partial volume effects due to the increased voxel volume (∼2.5‐fold), inferior point spread function and effects of the Hamming Filter. Interestingly, significant differences in regional ^2^H‐Glc uptake between GM and WM tissue was also observed in lower resolution PE‐DMI maps. This can be explained as voxel‐wise GM segmentation in the cerebral cortex for low spatial resolution is not feasible and voxels with GM fractions of >55% are predominantly located in the cerebellar cortex, where partial volume contamination affects results to a lesser degree. Therefore, the segmentation threshold for GM and WM regions initially was lowered to 55% for PE‐DMI, to increase the number of voxels in the analysis for a fair comparison with higher resolution CRT‐DMI.

In principle, this applies similarly for averaged Glx concentrations using identical GM masks, but lower cerebellar Glx/tCr ratios have been reported (Goryawala et al., 2016), which could explain, why metabolic differences between GM and WM could not be reproduced using PE‐DMI.

In general, MRSI datasets feature high redundancy and additional spectral sparsity of deuterium spectra are suited particularly well for low‐rank denoising during postprocessing (Clarke & Chiew, 2022; Nguyen et al., 2013).

Low‐rank approaches have been shown to reduce measurement uncertainty (Clarke & Chiew, 2022), and although its estimation via residual baseline noise, for example, SNR calculations or quality criteria threshold using CRLBs, is insufficient given the possibility of non‐uniform variance, we chose a relatively high threshold of 50% to exclude outliers. Additionally, it is not always recommended using relative CRLBs to estimate the standard deviation and exclude data points according to a strict threshold, especially when dealing with low SNR data (Kreis, 2016; Landheer & Juchem, 2021). SNR comparisons were used to illustrate the denoising of spectra and should not be used as a quantitative measure for comparison with other studies. Improved precision and accuracy to predict metabolite concentrations compared with the simulated gold standard has been shown after low‐rank denoising of synthetic data with a similar noise level compared with in vivo datasets. The fact, that averaged time courses of the gold standard presented from synthetic data (featuring small standard deviations; see Figure S4) is caused by the partial volume effects due to down‐sampling of high‐resolution data in k‐space and 3D Hamming filtering. This causes significant partial volume effects leading to signal blurring for DMI data. The same applies for segmentation masks of synthetic gray (sGM) and white matter (sWM), which is why a 70% threshold was used. Although in vivo data showed increasing deuterium labeled water concentrations over time, synthetic data featured time invariant water concentrations to test the performance of the low rank denoising algorithm, while not introducing a dynamic behavior for static metabolite concentrations.

Although the spatial resolution was increased by 2.5‐fold the point spread function is still suboptimal and significant partial volume errors are still the main limitations of the proposed ^2^H‐CRT‐DMI method. While further increasing the spatial resolution is theoretically feasible, and could potentially further improve the detection of regional variation of metabolic activity, inherently low SNRs are severely limiting the minimum achievable voxel size. Compressed sensing reconstruction approaches using *k*–*t* undersampling could further increase the sensitivity and the spatial resolution (Lingala et al., 2011; Lustig et al., 2007; Tsao et al., 2003). Similar to the low‐rank spatiotemporal denoising as performed in this study, k‐t undersampling rely on acquisition of redundant data over the whole measurement time, to efficiently denoise data of each time point. In general, DMI acquisitions do not necessarily require a baseline scan and could be performed as a “single‐shot” measurement, which would significantly reduce the overall MR scan time to roughly ∼15 min, but assessing the dynamics of glucose metabolism over a longer period of time allows for deriving potentially meaningful biomarkers, that is, metabolic fluxes and turnover rates. Additionally, to perform concentration estimation in millimolar, natural abundance water concentrations need to be acquired, at best, before or shortly after administration of deuterium‐labeled glucose, as the water signal increases over time as a result of deuterium uptake into the water pool. Altogether, the benefits of a dynamic assessment of deuterium labeled substrates over a longer period of time might outweigh the advantage of reduced acquisition time. No increase of the natural abundance ^2^H water signal was expected in the first 30 min after oral deuterium labeled glucose administration (Niess, Strasser, et al., 2023), therefore no correction was applied.

Other techniques such as multiecho steady state free precession DMI (Montrazi et al., 2023; Peters et al., 2021) or deep learning approaches (Li et al., 2021) could also significantly increase the achievable SNR and consequently the spatial resolution.

In principle lactate detection is feasible using DMI and has been shown in tumor patients (De Feyter et al., 2018), but in the healthy human brain only 5% of metabolized glucose undergoes anaerobic glycolysis leading to lactate production and additionally could be built up via pyruvate/lactate exchange. However, apparent lactate concentrations in the healthy human brain are below the detection limit of the applied method, even when regional averaging over multiple voxels is performed. One of the main advantages of direct deuterium detection using DMI compared with indirect deuterium detection using QELT is the whole‐brain coverage, due to unlocalized excitation compared with slice‐selective excitation used in ^1^H‐MRSI. This allows for analyzing multiple regions of interest in the brain simultaneously. This could be beneficial for several pathologies where multiple pathologic regions occur, for example, MS or during functional studies using stimulation, for example, fMRSI.

## 5 CONCLUSION

This study demonstrated the feasibility of dynamic whole‐brain deuterium metabolic imaging to reproduce reported regional differences in kinetics of glucose metabolism between gray and white matter tissue in the human brain. 3D metabolic maps of labeled glucose and downstream neurotransmitters, that is, combined glutamate and glutamine were acquired dynamically every 7 min with sub‐milliliter isotropic spatial resolution. This suggests significant potential for clinical applications with improved characterization of local pathologic alterations and for monitoring the dynamics of glucose metabolism with increased specificity in a completely non‐invasive way.

## FUNDING INFORMATION

This work was supported by the National Institute of Health NIH R01EB031787, the Austrian Science Fund: WEAVE I 6037, KLI 1106 & P36328‐N the Christian Doppler Laboratory for MR Imaging Biomarkers (BIOMAK) and the European Union (ERC, GLUCO‐SCAN, 101088351). WTC was funded by the Wellcome Trust [225924/Z/22/Z].

## CONFLICT OF INTEREST STATEMENT

R. Lanzenberger received investigator‐initiated research funding from Siemens Healthcare regarding clinical research using PET/MR. He is a shareholder of the start‐up company BM Health GmbH since 2019.

## INFORMED CONSENT

Written informed consent was obtained from all participants.

## Supporting information


**Figure S1.** Time courses of representative axial ^2^H glucose (Glc) maps given in mM from all participants, detected using deuterium metabolic imaging (DMI) with phase‐encoded readout (orange) and concentric ring trajectory readout (blue) at 7 T. Missing voxels in the metabolic maps do not contain a value. NaN, not a number.

**Figure S2.** Time courses of representative axial ^2^H glutamate + glutamine (Glx) maps given in mM from all participants, detected using deuterium metabolic imaging (DMI) with phase encoded readout (orange) and concentric ring trajectory readout (blue) at 7 T. Missing voxels in the metabolic maps do not contain a value. NaN, not a number.

**Figure S3.** Representative sample spectra and residues from synthetic data (a) without noise (Gold standard, GS), added noise to mimic SNR of in vivo data (NO), and following de‐noising using low‐rank approximation (LR). Three‐dimensional metabolic maps of glucose (Glc), glutamate + glutamine (Glx) and natural abundance water from synthetic data (b) for all three scenarios (GS, NO, LR).

**Figure S4.** Performance illustration of the applied low‐rank denoising approach. Time courses of deuterium‐labeled substrates from synthetic data without noise (gold standard), added noise, and following low‐rank denoising, averaged over gray (blue, GM) and white matter (red, WM) dominated regions. Glutamate + glutamine (Glx) was synthesized to increase strictly linearly over time with 43% higher concentrations in GM compared with WM for the last time point. Glucose increases monoexponentially with identical time constants in GM and WM (15 min) and 19% higher concentrations in GM compared with WM. Water concentrations are constant over time, while 10% higher concentrations were introduced in GM compared with WM, on average over all time points.

**Data S1.** Supporting Information.

## Data Availability

Data generated by postprocessing (i.e., metabolic maps, LCModel basis sets, script files for data plotting) are available from the corresponding author on reasonable request for research purposes only.
