# Supplementary figures and images for: Whole‐brain deuterium metabolic imaging via concentric ring trajectory readout enables assessment of regional variations in neuronal glucose metabolism
Source: Hum Brain Mapp. 2024 Apr 22;45(6):e26686. doi: 10.1002/hbm.26686 (PMC11034002; doi:10.1002/hbm.26686)

PE DMI  
16x16x14  
2.0 ml  
 $T_A = 7$  min

CRT DMI  
22x22x21  
0.75 ml  
 $T_A = 7$  min

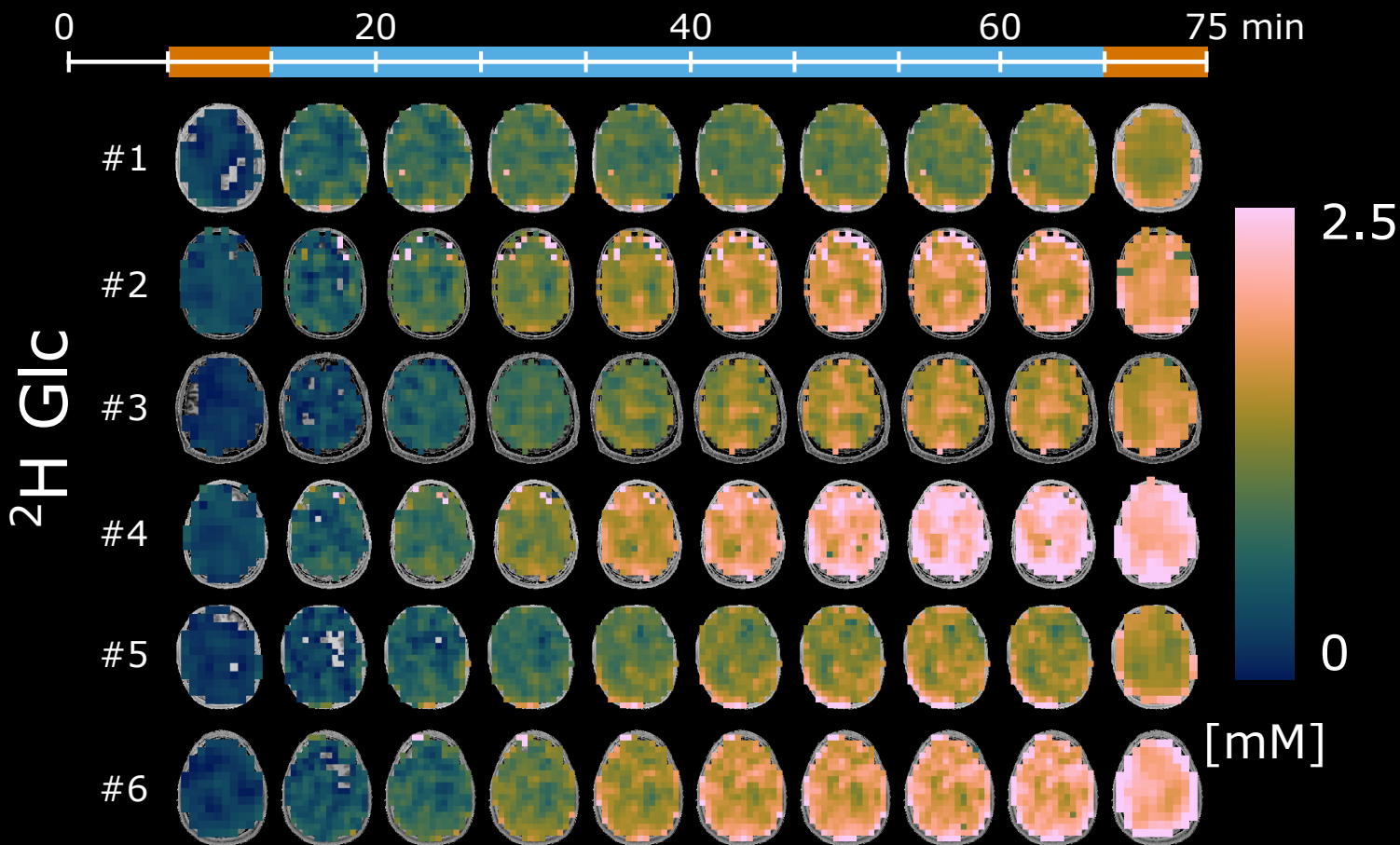

Supplement: Supplementary file 1 — Figure S1. Time courses of representative axial 2H glucose (Glc) maps given in mM from all participants, detected using deuterium metabolic imaging (DMI) with phase‐encoded readout (orange) and concentric ring trajectory readout (blue) at 7 T. Missing voxels in the metabolic maps do not contain a value. NaN, not a number. [file HBM-45-e26686-s002.pdf]

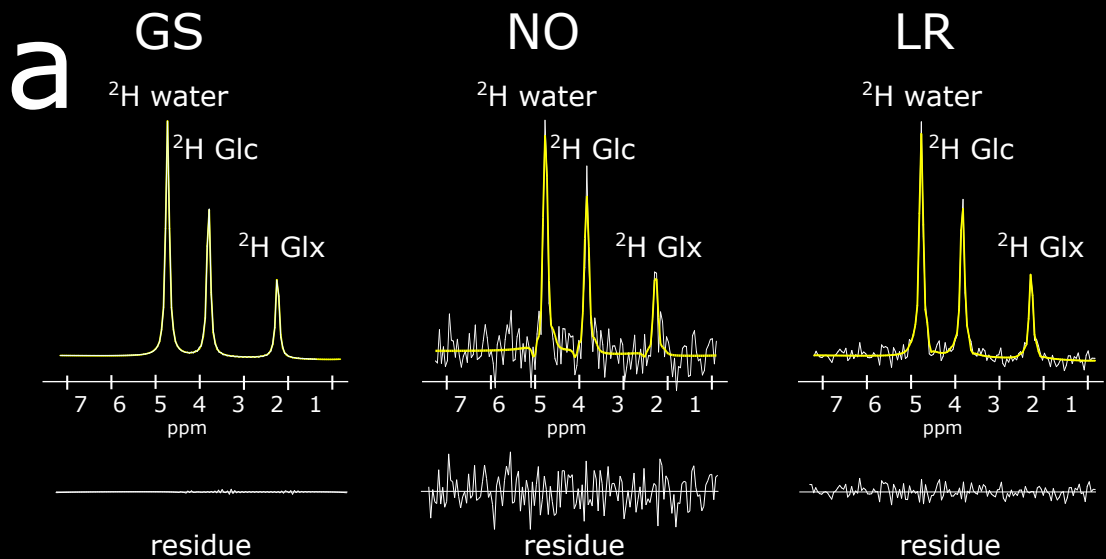

GS: Gold standard (no noise added)

NO: Noise added

LR: Noise added + Low rank denoising

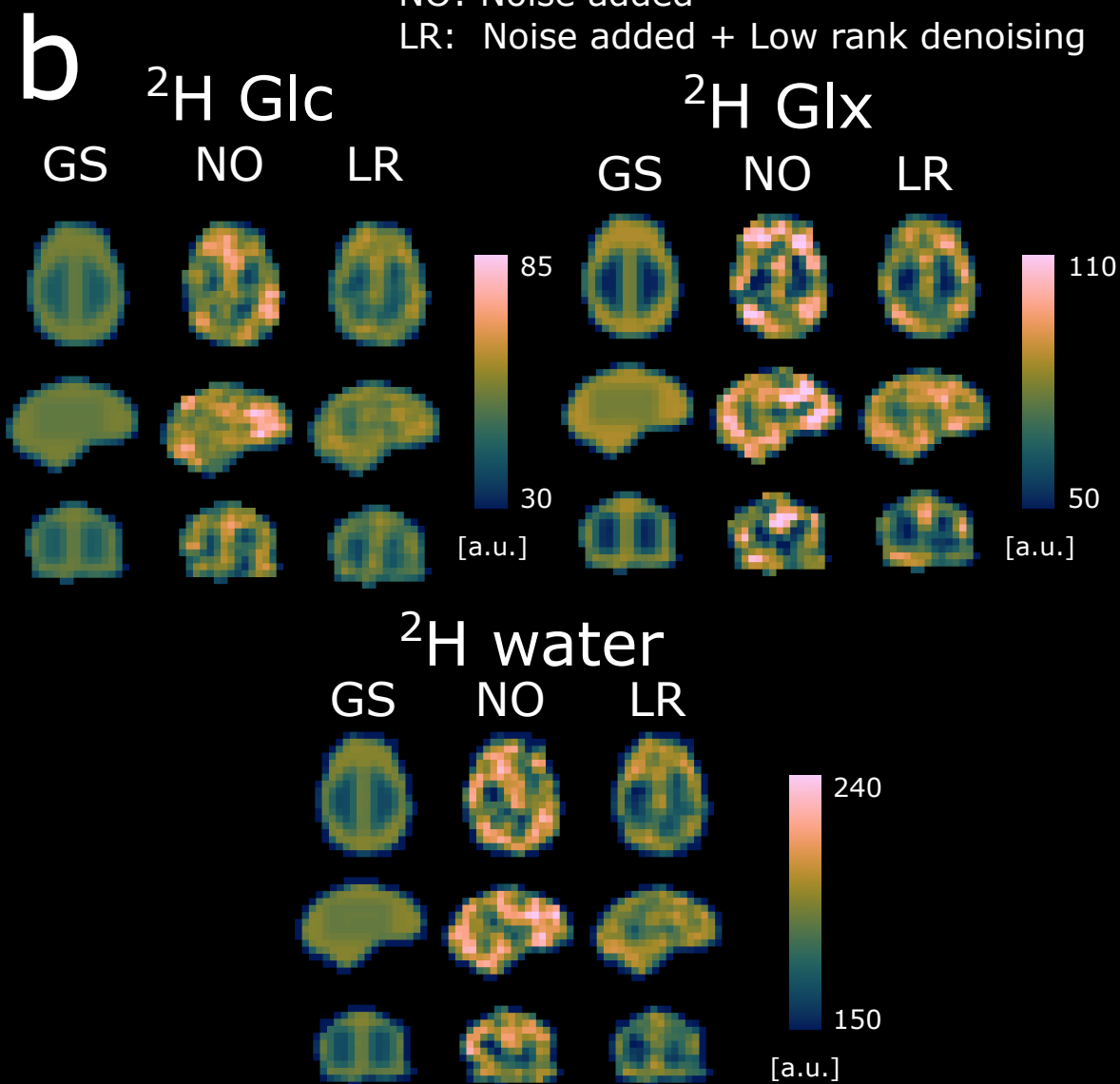

Supplement: Supplementary file 3 — Figure S3. Representative sample spectra and residues from synthetic data (a) without noise (Gold standard, GS), added noise to mimic SNR of in vivo data (NO), and following de‐noising using low‐rank approximation (LR). Three‐dimensional metabolic maps of glucose (Glc), glutamate + glutamine (Glx) and natural abundance water from synthetic data (b) for all three scenarios (GS, NO, LR). [file HBM-45-e26686-s003.pdf]

Gold standard  
(GS)

Noise added  
(NO)

De-noised  
(LR)

$^2\text{H Glc}$

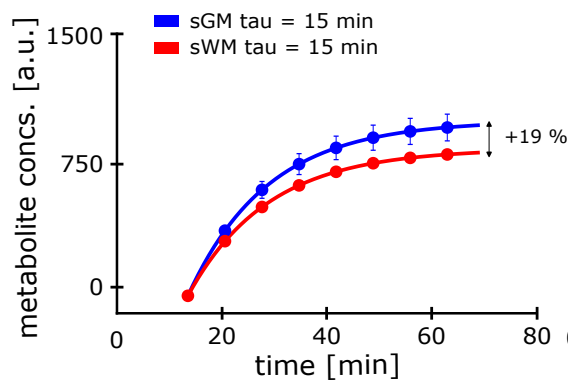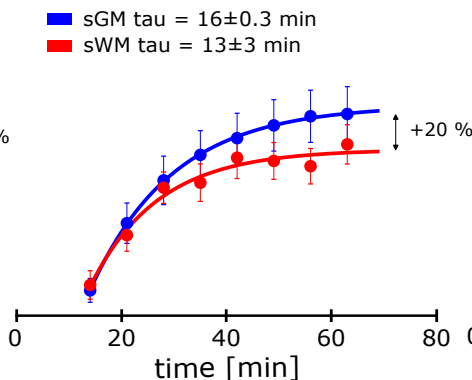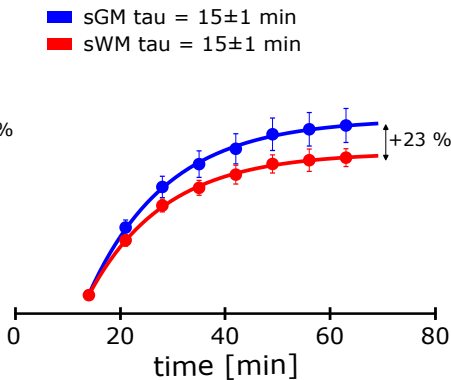

$^2\text{H Glx}$

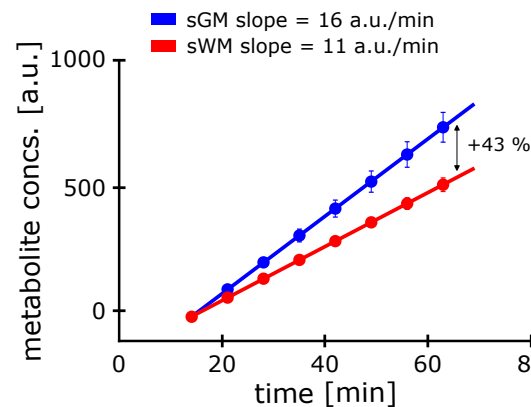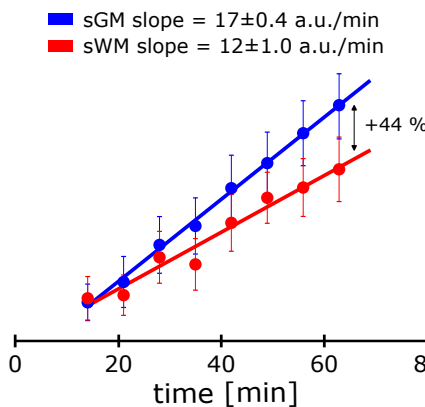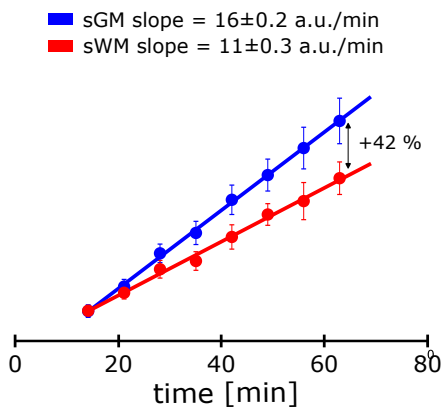

$^2\text{H water}$

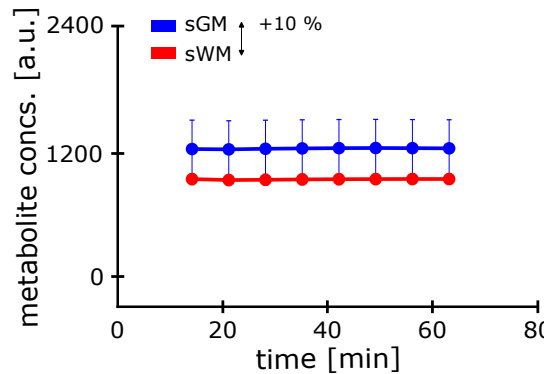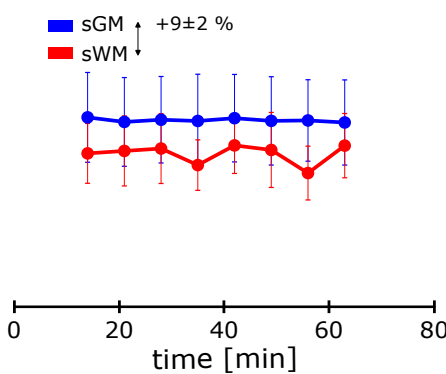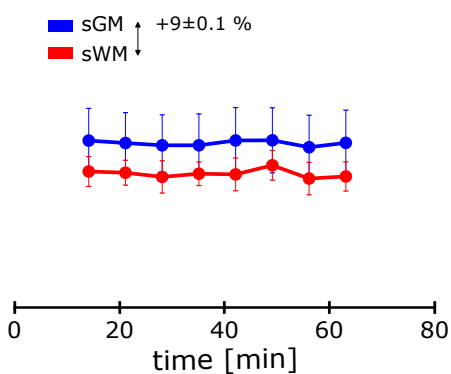

Supplement: Supplementary file 4 — Figure S4. Performance illustration of the applied low‐rank denoising approach. Time courses of deuterium‐labeled substrates from synthetic data without noise (gold standard), added noise, and following low‐rank denoising, averaged over gray (blue, GM) and white matter (red, WM) dominated regions. Glutamate + glutamine (Glx) was synthesized to increase strictly linearly over time with 43% higher concentrations in GM compared with WM for the last time point. Glucose increases monoexponentially with identical time constants in GM and WM (15 min) and 19% higher concentrations in GM compared with WM. Water concentrations are constant over time, while 10% higher concentrations were introduced in GM compared with WM, on average over all time points. [file HBM-45-e26686-s004.pdf]
