# Supplementary material for: Whole‐brain deuterium metabolic imaging via concentric ring trajectory readout enables assessment of regional variations in neuronal glucose metabolism
Source: Hum Brain Mapp. 2024 Apr 22;45(6):e26686. doi: 10.1002/hbm.26686 (PMC11034002; doi:10.1002/hbm.26686)
Supplement: Supplementary file 2 — Figure S2. Time courses of representative axial 2H glutamate + glutamine (Glx) maps given in mM from all participants, detected using deuterium metabolic imaging (DMI) with phase encoded readout (orange) and concentric ring trajectory readout (blue) at 7 T. Missing voxels in the metabolic maps do not contain a value. NaN, not a number. [file HBM-45-e26686-s001.pdf]

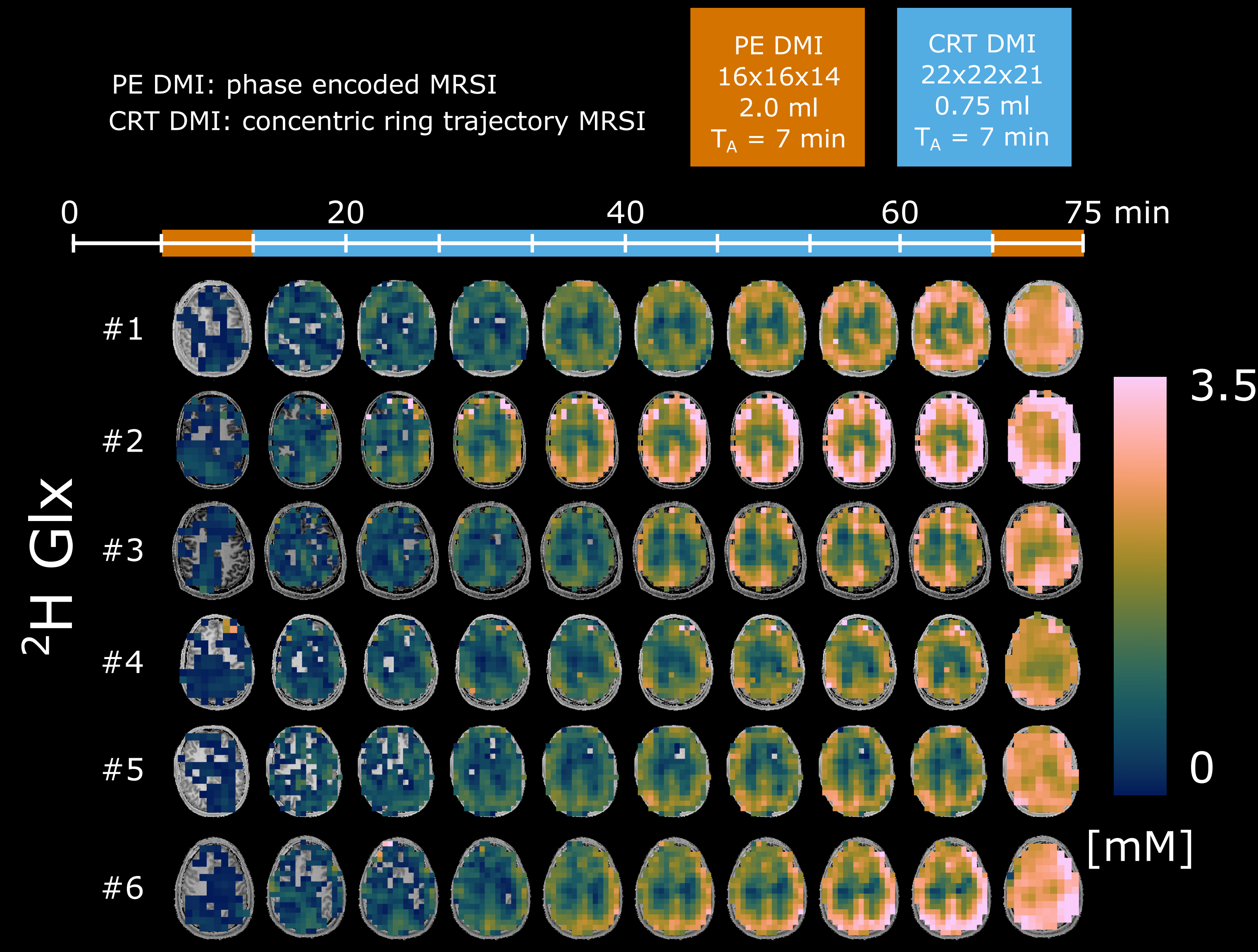

PE DMI: phase encoded MRSI
CRT DMI: concentric ring trajectory MRSI
PE DMI
16x16x14
2.0 ml
T_A = 7 min
CRT DMI
22x22x21
0.75 ml
T_A = 7 min
0
20
40
60
75 min
^{2}H Glx
#1
#2
#3
#4
#5
#6
3.5
0
[mM]
