# Supplementary material for: Whole‐brain deuterium metabolic imaging via concentric ring trajectory readout enables assessment of regional variations in neuronal glucose metabolism
Source: Hum Brain Mapp. 2024 Apr 22;45(6):e26686. doi: 10.1002/hbm.26686 (PMC11034002; doi:10.1002/hbm.26686)
Supplement: Supplementary file 5 — Data S1. Supporting Information. [file HBM-45-e26686-s005.docx]

# Supplementary material

|  |  |  | |  |
| --- | --- | --- | --- | --- |
| *Minimum Reporting Standards in MR Spectroscopy checklist (according to Lin et al. NMR Biomed 2021)* | | | | |
| **1. Hardware** | | |  | |
| *a. Field strength [T]* | | | 7T | |
| *b. Manufacturer* | | | Siemens | |
| *c. Model (software version if available)* | | | Magnetom dot Plus | |
| *d. RF coils: nuclei (transmit/ receive), number of channels, type, body part* | | | ^2^H/^1^H dual tuned quadrature birdcage head coil, transmit/receive, 1 channel, (Stark Contrast MRI Coils Research, Germany) | |
| *e. Additional hardware* | | | N/A | |
| **2. Acquisition** | | |  | |
| *a. Pulse sequence* | | | 3D FID-acquire elliptical phase encoding (PE) MRSI & 3D FID-acquire density-weighted concentric ring trajectory (CRT) MRSI | |
| *b. Volume of Interest (VOI) locations* | | | whole-brain, unlocalized excitation using rectangular RF pulse with 86° flip angle | |
| *c. Nominal VOI size [cm^3^, mm^3^]* | | | PE MRSI: (FOV) 200x200x175 mm^3^  CRT MRSI: FOV 200x200x192 mm^3^ | |
| *d. Repetition Time (TR), Echo Time (TE) [ms, s]* | | | PE MRSI: TR=290 ms / 1.56 ms acquisition delay  CRT MRSI: TR = 290 ms / 2 ms acquisition delay | |
| *e. Total number of Excitations or acquisitions per spectrum* | | | PE MRSI 2 averages (7 min acquisition time)  CRT MRSI: 44 circles (matched 7 min acquisition times) | |
| *In time series for kinetic studies* | | | N/A | |
| *i.         Number of Averaged spectra (NA) per time-point* | | | N/A | |
| *ii.       Averaging method (e.g. block-wise or moving average)* | | | N/A | |
| *iii.      Total number of spectra (acquired / in time-series)* | | | N/A | |
| *f. Additional sequence parameters (spectral width in Hz, number of spectral points, frequency offsets); If STEAM: Mixing Time TM; If MRSI: 2D or 3D, FOV in all directions, matrix size, acceleration factors* | | | PE MRSI: BW: 500 Hz, 128 spectral points,16x16x14  CRT MRSI: BW: 380 Hz, 96 spectral points, 22x22x21 | |
| *g. Water Suppression Method* | | | No water suppression | |
| *h. Shimming Method, reference peak, and thresholds for “acceptance of shim” chosen* | | | Standard shim + manual adjustment, ^1^H water peak < 40 Hz, ^2^H water peak < 30 Hz Region: whole brain | |
| *i. Triggering or motion correction method (respiratory, peripheral, cardiac triggering, incl. device used and delays)* | | | - | |
| **3. Data analysis methods and outputs** | | |  | |
| *a. Analysis software* | | | LCModel 6.3-1 | |
| *b. Processing steps deviating from quoted reference or product* | | | N/A | |
| *c. Output measure (e.g. absolute concentration, institutional units, ratio)* | | | concentration estimation in mM | |
| *d. Quantification references and assumptions, fitting model assumptions* | | | Simulated in NMRScope-B | |
| **4. Data Quality** | | |  | |
| *a. Reported variables (SNR, Linewidth (with reference peaks))* | | | SNR was calculated using voxel-wise signal maximum of water / sd of noise 200 Hz off-center | |
| *b. Data exclusion criteria* | | | CRLBs > 50 % for water, Glc, Glx, no CRLB threshold for first 3 time points (first 20min) | |
| *c. Quality measures of postprocessing Model fitting (e.g. CRLB, goodness of fit, SD of residual)* | | | CRLB | |
| *d. Sample Spectrum* | | | See Figure 5 | |

**Supplementary Table 1:**

Minimum Reporting Standards for in vivo MR Spectroscopy

Note. – Parameters 7T DMI**,** CRLB = Cramér-Rao lower bounds; FID = free induction decay; CRT = concentric ring trajectory; FOV = field of view; FWHM = full-width-at-half-maximum; Glx = Glutamate+Glutamine; Glc = Glucose; SNR = signal-to-noise ratio; VOI = volume of interest.

**^2^H DMI:**

$$\left[ \boldsymbol{M}_{\boldsymbol{Abs}} \right]\boldsymbol{=}\frac{\boldsymbol{Amplitude}_{\boldsymbol{M}}}{\boldsymbol{Amplitude}_{\boldsymbol{Water}}}\boldsymbol{*}\frac{\boldsymbol{f}_{\boldsymbol{GM}}\boldsymbol{*}\boldsymbol{d}_{\boldsymbol{GM}}\boldsymbol{*}\boldsymbol{R}_{\boldsymbol{wate}\boldsymbol{r}_{\boldsymbol{GM}}}\boldsymbol{+}\boldsymbol{f}_{\boldsymbol{WM}}\boldsymbol{*}\boldsymbol{d}_{\boldsymbol{WM}}\boldsymbol{*}\boldsymbol{R}_{\boldsymbol{wate}\boldsymbol{r}_{\boldsymbol{WM}}}\boldsymbol{+}\boldsymbol{f}_{\boldsymbol{CSF}}\boldsymbol{*}\boldsymbol{d}_{\boldsymbol{CSF}}\boldsymbol{*}\boldsymbol{R}_{\boldsymbol{wate}\boldsymbol{r}_{\boldsymbol{CSF}}}}{\left( \boldsymbol{1}\boldsymbol{-}\boldsymbol{f}_{\boldsymbol{CSF}} \right)\boldsymbol{*}\boldsymbol{R}_{\boldsymbol{M}}}\boldsymbol{*}\boldsymbol{17}\boldsymbol{.}\boldsymbol{2}\boldsymbol{mM}\boldsymbol{*}\frac{\boldsymbol{N}_{\boldsymbol{water}}}{\boldsymbol{N}_{\boldsymbol{M}}}\boldsymbol{*}\frac{\boldsymbol{1}}{\boldsymbol{1-0.4}}$$

$$\boldsymbol{R}_{\boldsymbol{M}}\boldsymbol{=}\boldsymbol{e}^{\boldsymbol{-}\boldsymbol{T}_{\boldsymbol{E}}\boldsymbol{/}\boldsymbol{T}_{\boldsymbol{2}}}\boldsymbol{*(1-}\boldsymbol{e}^{\boldsymbol{-}\boldsymbol{T}_{\boldsymbol{R}}\boldsymbol{/}\boldsymbol{T}_{\boldsymbol{1}}}\boldsymbol{)}$$

**Appendix 1:**

Concentration estimation in mM units of ^2^H resonances detected using DMI at 7T. Metabolite amplitudes (${Amplitude}_{M}$) were referenced to deuterated water signals (${Amplitude}_{Water}$) and corrected for relaxation times $R_{M}$ and voxel-wise fractional water content for GM and WM and CSF tissue ($f_{GM},f_{WM}$) with $d_{GM}$=0.78, $d_{WM}$=0.65 and $d_{CSF}$=0.97.

For concentration estimation of glucose, the term (1-f_CSF_) was excluded.

Correction for ^2^H label loss of Glx was performed according to de Graaf et al. 2021 (assuming on average approximately 40%.

**
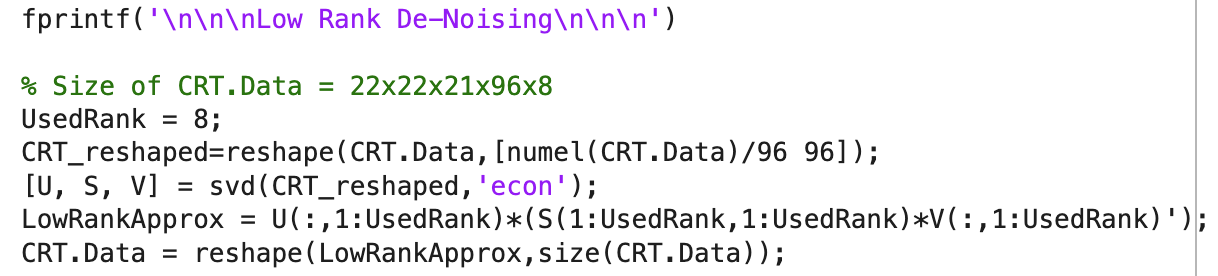
**

**Appendix 2:**

Low rank spatio-temporal denoising using singular value decomposition algorithm.

Prior, data (22x22x21: 3 spatial dimensions, 96 spectral points, 8 repetitions/time points) was reshaped into a 2D structure before performing SVD using the in-built MATLAB function. Low rank approximation was performed using a fixed rank (8) followed by reshaping to the initial matrix dimensions.

**
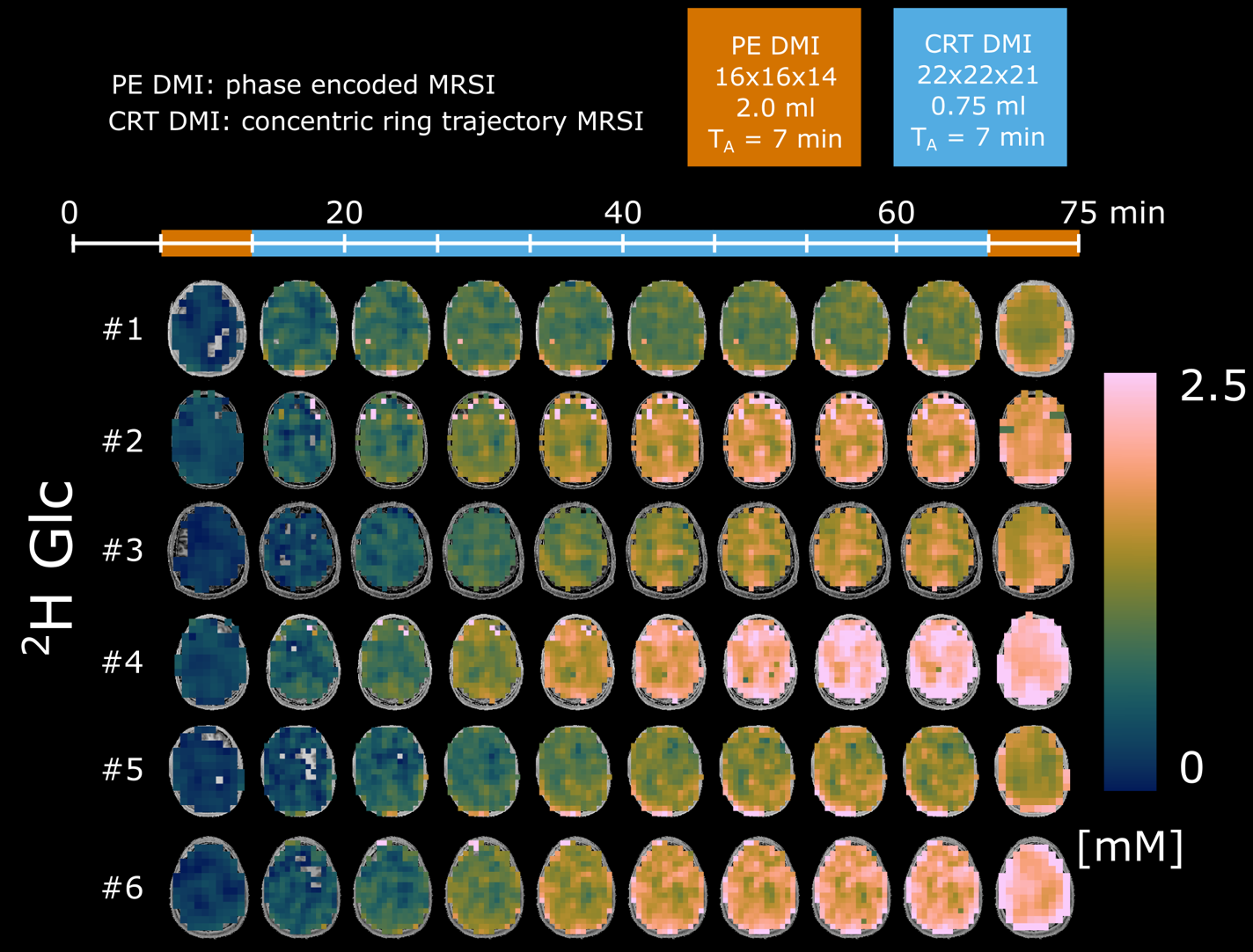
**

**Supplementary Figure 1:** Time courses of representative axial ^2^H glucose (Glc) maps given in mM from all participants, detected using deuterium metabolic imaging (DMI) with phase encoded readout (orange) and concentric ring trajectory readout (blue) at 7T. Missing voxels in the metabolic maps do not contain a value (NaN: not a number).


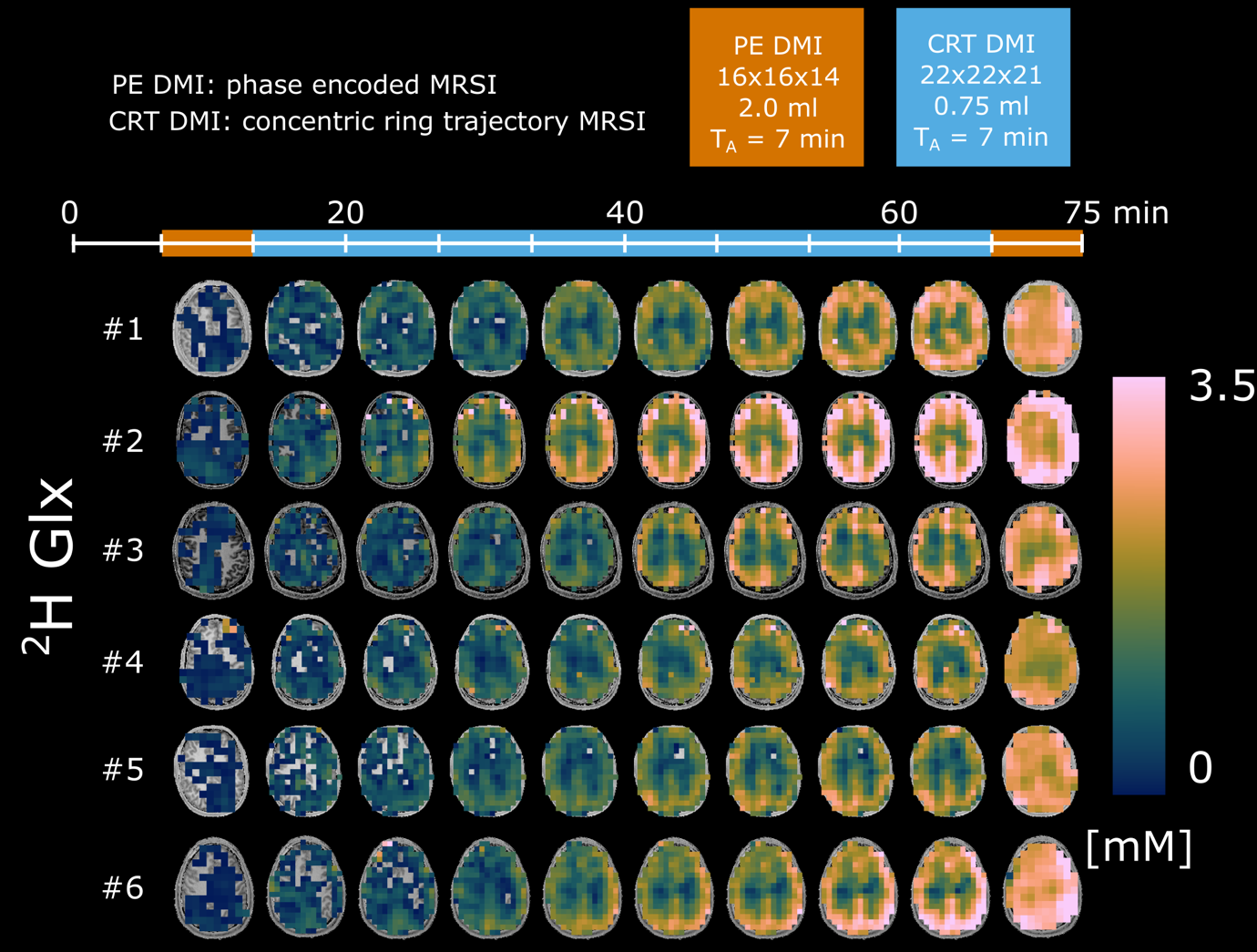


**Supplementary Figure 2:** Time courses of representative axial ^2^H glutamate+glutamine (Glx) maps given in mM from all participants, detected using deuterium metabolic imaging (DMI) with phase encoded readout (orange) and concentric ring trajectory readout (blue) at 7T. Missing voxels in the metabolic maps do not contain a value (NaN: not a number).


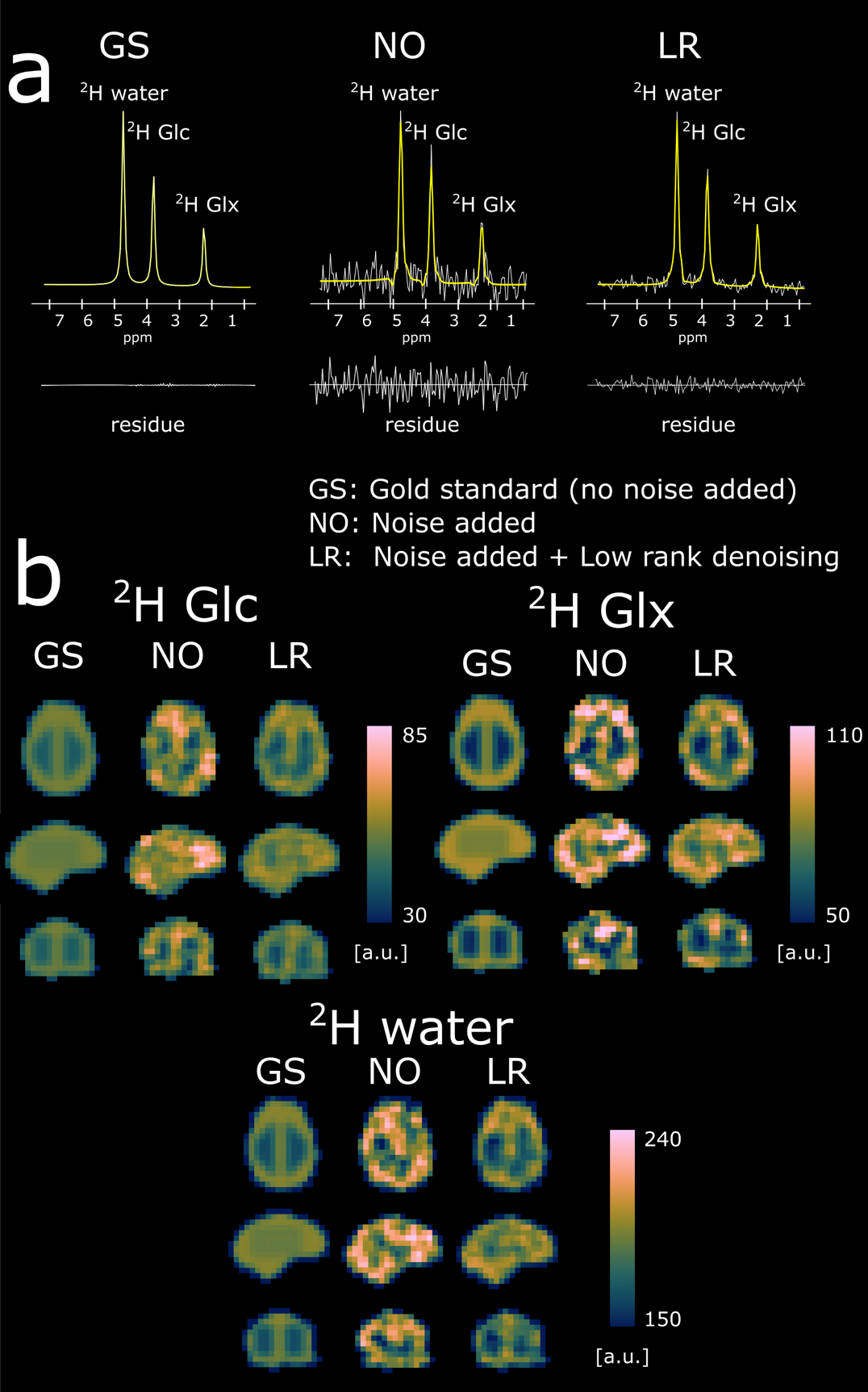


**Supplemental Figure 3:** Representative sample spectra and residues from synthetic data (a) without noise (Gold standard, GS), added noise to mimic SNR of in vivo data (NO), and following de-noising using low rank approximation (LR). 3D Metabolic maps of glucose (Glc), glutamate+glutamime (Glx) and natural abundance water from synthetic data (b) for all three scenarios (GS, NO, LR).

**
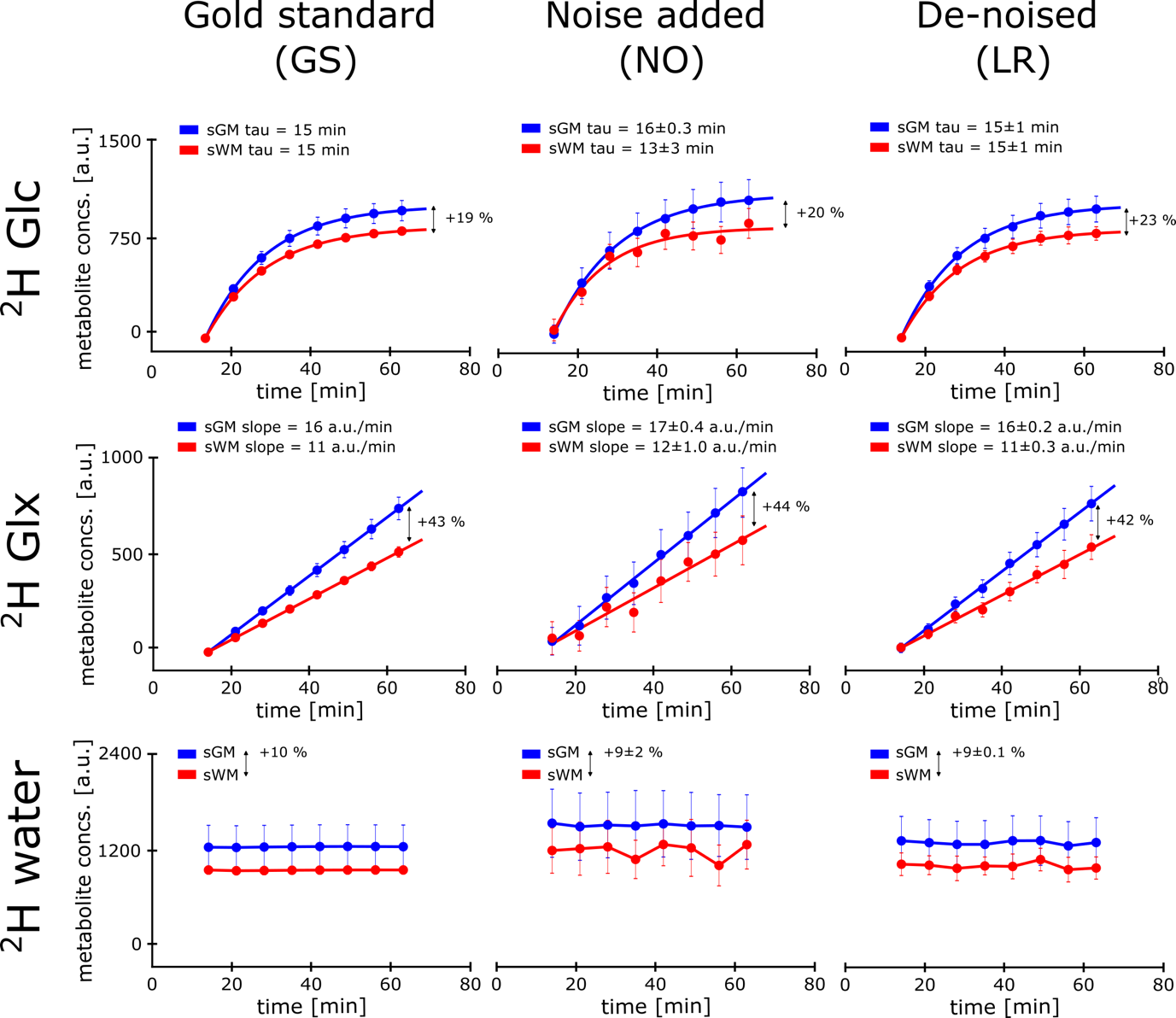
Supplementary Figure 4:**

Performance illustration of the applied low rank denoising approach. Time courses of deuterium labeled substrates from synthetic data without noise (gold standard), added noise, and following low-rank denoising, averaged over gray (blue, GM) and white matter (red, WM) dominated regions. Glutamate+glutamine (Glx) was synthesized to increase strictly linearly over time with 43% higher concentrations in GM compared to WM for the last time point. Glucose increases mono-exponentially with identical time constants in GM and WM (15 min) and 19% higher concentrations in GM compared to WM. Water concentrations are constant over time, while 10% higher concentrations were introduced in GM compared to WM, on average over all time points.
